# Isolation, purification, and phenotypic characterization of virulent *Klebsiella pneumoniae phages* from environmental samples in Addis Ababa, Ethiopia: A synergistic approach combining spot assay and streak plating

**DOI:** 10.1371/journal.pone.0331955

**Published:** 2025-09-24

**Authors:** Assefa Asnakew Abebe, Alemayehu Godana Birhanu, Tesfaye Sisay Tessema

**Affiliations:** 1 Unit of Molecular Biology and Bioinformatics, Institute of Biotechnology, Addis Ababa University, Addis Ababa, Ethiopia; 2 Department of Medical Laboratory Sciences, Institute of Health, Bule Hora University, Bule Hora, Ethiopia; University of Gondar College of Medicine and Health Sciences, ETHIOPIA

## Abstract

**Background:**

Multidrug-resistant (MDR) *Klebsiella pneumoniae* poses a significant global health threat, necessitating alternative therapies like bacteriophage treatment. Traditional phage isolation methods, such as plaque assays, are resource-intensive and thus limit large-scale screening. This study aimed to isolate and purify phages targeting MDR *K. pneumoniae* using a combined spot-streak plating method and to compare its efficiency with conventional techniques.

**Materials and methods:**

A total of 62 environmental samples were screened against two MDR *K. pneumoniae* isolates using a combined approach: initial phage detection by spot assays followed by streak plate-based purification for isolating pure phage clones. Traditional plaque assays were also performed for comparison. Isolated phages were characterized by performing host range analysis against 45 bacterial isolates and determining their burst size. Additionally, their stability was assessed across various pH and temperature conditions, and single-step growth curve experiments were conducted.

**Results:**

The combined spot assay and streak plate purification method yielded 22 phages, whereas plaque assays isolated 17 phages. Eight phages with high titers and lytic activity were selected for further analysis. These phages exhibited broad host ranges, with two phages lysing up to 55.5% of tested isolates. Stability assays showed effective lytic activity between pH 5 and 9 and temperatures up to 60°C. Single-step growth curves revealed latent periods ranging from 15 to 40 minutes and burst sizes between 76 and 310 PFU per infected cell. The combined method reduced both the isolation time and resource requirements.

**Conclusion:**

The integration of spot assays with streak plate-based purification provides an efficient and less resource-intensive method for isolating diverse phages targeting MDR K. pneumoniae. The isolated phages demonstrate promising broad-spectrum lytic activity and physiological stability, supporting their potential use in phage therapy.

**Recommendation:**

Further genomic characterization is necessary to confirm the strictly lytic nature of these phages and to exclude the presence of undesirable genes. Subsequent studies should focus on in vivo efficacy testing and formulation optimization to facilitate clinical application.

## 1. Introduction

The emergence of antimicrobial resistance (AMR) has become a global health challenge, prompting the World Health Organization (WHO) to prioritize it as a critical public health threat [1,2]. Among the pathogens prioritized by WHO in the fight against AMR, *K. pneumoniae* stands out as a major concern [3]. *K. pneumoniae* causes various infections, with urinary tract infections, pneumonia, and sepsis being particularly common [4,5]. These infections can occur in both community and healthcare settings [6].

The increasing prevalence of MDR *K. pneumoniae* has skyrocketed, necessitating the search for an alternative approach [7,8]. One promising approach is phage therapy, which utilizes lytic bacterial viruses [9–11]. Bacteriophage therapy involves the use of bacteriophages (phages) to treat bacterial infections, and is being utilized as an alternative therapeutic option for drug-resistant infections caused by this pathogen. Phages are viruses that infect and lyse bacterial cells specifically. Because of their specificity, they are a preferred approach in combating infections caused by bacteria [12]. Bacteriophages are highly specific viruses that target bacteria. Phages are found in various natural environments and have shown promise as an alternative to antibiotics [13,14]. Isolating and purifying phages is the essential step in their investigation and application. It is particularly crucial for phage biology research and therapeutic [15].

Isolating multiple phages is crucial when developing effective phage therapies to combat AMR [16–19]. By isolating different types of phages, we can create cocktails that efficiently target multiple bacterial strains or species. This is vital in clinical settings where various pathogens may cause infections, necessitating a broad-spectrum therapeutic approach or the isolation of specific phages for targeted therapy. Bacteria can develop resistance to phages [20–22]. All phages must be isolated and purified before they can be investigated or utilized for biological characterization and application, making phage isolation a crucial step. Isolating a variety of phages is crucial for several reasons, especially when developing efficient phage therapies to fight antibiotic resistance (AMR) [17,23].

The emergence of phage-resistant bacterial strains, which hinders single phage therapy, underscores the need for a diverse array of phages. Therefore, isolating various phages that can target different strains of bacteria is crucial for developing robust phage cocktails that increase therapeutic efficacy while reducing the likelihood of phage resistance. This flexibility is judged mandatory for phage therapy success, especially in cases of recurrent or chronic infections where pathogens may eventually become resistant [24–26].

The primary steps in developing phage-based applications, including phage therapy, involve isolating and purifying lytic phages. However, current methods and protocols for phage isolation face significant challenges in being complex, time-consuming, and resource-intensive [27]. These challenges hinder the efficient isolation and purification of phages from many samples within a short time, especially in on-demand urgent phage isolation, which is necessary for developing phage cocktails that can target different bacterial strains. While the plaque assay method is widely used, it requires significant resources and time to isolate and purify individual phages. This process often involves picking plaques, suspending them in a buffer, purifying the lysate the following day, performing serial dilutions, and testing each dilution on a single host. This procedure can take 4–5 days to isolate and purify a single phage. To address these limitations, some researchers have explored using the spot assay for phage isolation [28].

The spot assay allows for rapid screening of many potential phage-containing samples against a single bacterial host in a single experiment. However, the downstream purification of isolated phages using the spot assay still relies on plaque assay methods, which can be tedious and labor-intensive [15,29,30]. Efficient phage isolation is significant because phages have the potential to serve as adjunct or alternative to antibiotics. The development of phage cocktails further emphasizes the need for rapid and reliable methods to isolate novel phages. While some researchers have used phage streak plating to directly detect phages in samples [31–33], these methods still rely on plaque assays for purification.

In contrast, the current study uses spot assay to efficiently recover *K. pneumoniae* phages and introduces streak plating as a purification technique. This study combined spot assay with a unique streak-plating method for phage isolation and purification. The approach saves time, resources, and labor, and allows for the screening of multiple samples with limited resources, which is advantageous in resource-limited areas.

Instead of traditional plaque assay methods – such as suspending phages in buffer, conducting titrations, and performing serial dilutions- the developed method directly produces individually separated phage plaques from the lysis zone or plaques observed in spot plates. This method can also be applied to heat-sensitive hosts. This study aims to isolate and purify phages targeting MDR *K. pneumoniae* from various environmental sources using a spot assay for phage recovery and a plating technique for purification. Additionally, the study characterizes the isolated phages by examining their titer, host range, efficiency of plating, multiplicity of infection, burst size, latent period, and stability across different pH and temperature conditions.

## 2. Materials and methods

### 2.1. Study area and design

A cross-sectional study design was conducted in selected areas of Addis Ababa from February to November 2024. The laboratory work was conducted at the Health Biotechnology Laboratory, Institute of Biotechnology, Addis Ababa University. Addis Ababa is the capital and largest city of Ethiopia, located on a well-watered plateau surrounded by hills and mountains at an altitude of about 2,500 meter above sea level. The average annual temperature and rainfall are 21^o^C and 1800 mm, respectively.

**Fig 1:**
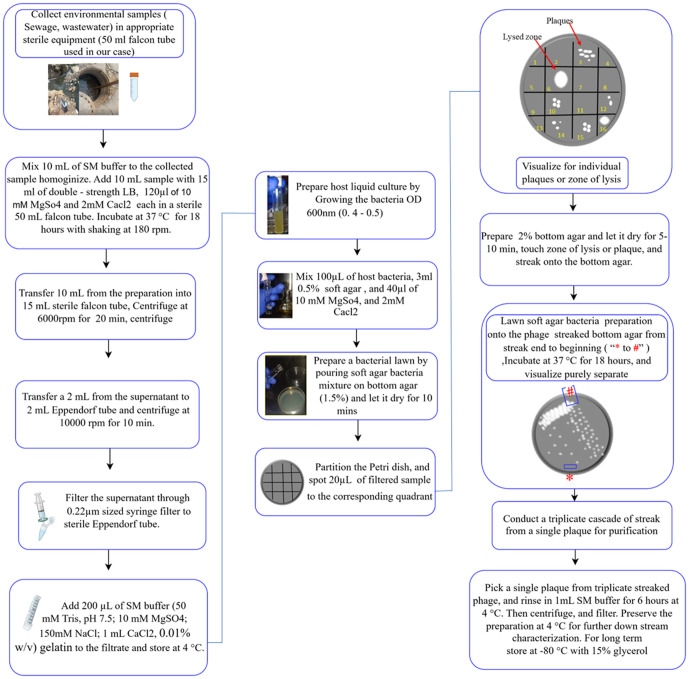
Schematic representation of the overall method from sample processing up to phage purification.

A convenient non-probability purposive sampling approach was used to select both the sampling sites and determine the sample sizes.Environmental sites-including hospital sewages, wastewater, and soils, were purposively chosen based on their high levels of organic disposal and waste contamination, conditions known to favor the presence and proliferation of bacteriophages. A total of 62 samples were collected from twenty sampling sites. The sampling sites and the type of samples collected can be found in (S1 Table). Samples were then assayed for bacteriophage recovery. The steps involved in sample processing, phage isolation, and purification are illustrated in (Fig 1).

#### 2.1.1. Collection and processing of wastewater and sewage samples.

Sewage and wastewater samples were collected and processed according to the method described in [34] with little modification. Two methods were used: a surface discrete sample taken from the top layer, and a mixed sample (by mixing samples from 25 cm and 45 cm below the surface). Forty milliliters of sample were collected using a 50 mL capped Falcon tube, labeled properly with the date, location, and type of collected sample, and transported to Addis Ababa University, Institute of Biotechnology, Health Biotechnology Laboratory in an ice box. Collected samples were retained at 4°C until further processing.

Saline Magnesium (SM) buffer (2 mM CaCl2, 10 mM MgSO4, 50 mM Tris-HCl pH 7.5, 150 mM NaCl, and 0.01% (w/v) gelatin) was used for sample homogenization and processing. One milliliter of SM buffer was added to the collected samples before processing, and homogenized by inverting 10–15 times. Ten milliliters from the homogenized sample were mixed with 15 mL double-strength Luria Broth (LB) (HIMEDIA, India) in a 50 mL capped Falcon tube and incubated at 37°C with shaking at 180 rpm for 18 hours, relying on endogenous bacteria for any phage amplification during incubation. Ten milliliters from the preparation were transferred to a 15 mL Falcon tube and centrifuged at 6000 rpm for 20 minutes, as illustrated in (Fig 1). Immediately after centrifugation, 2 mL of the supernatant was transferred to a 2 mL Eppendorf tube and centrifuged at 10,000 rpm for 10 minutes. Then the supernatant was filtered through a 0.22 µm-sized syringe filter to remove bacterial cells. The filtrate was retained at 4°C for further phage screening.

#### 2.1.2. Collection and processing of soil samples.

Soil samples were collected and processed according to the method described in [35]. Twenty grams of soil sample at a depth of 5–25 cm were collected in a sterile plastic bag from each site and transported to the Addis Ababa University Institute of Biotechnology Health Biotechnology Laboratory in an ice box. The collected samples were retained at 4°C until further processing. Ten grams of soil were added to 50 mL of double-strength Luria Broth (LB) (HiMedia, India), and 100 μL of SM buffer in a 250 mL sterile flask, and homogenized by thoroughly shaking 10–15 times. The preparation was incubated at 37°C for 18 hours with shaking at 180 rpm. Ten milliliters were transferred to 15 mL capped Falcon tubes and centrifuged at 6000 rpm for 20 minutes. From this step onwards, the same procedure was applied as in the sewage and wastewater sample processing illustrated in (Fig 1).

### 2.2. Host strain and growth conditions

The study included a total of 45 bacterial isolates, of which 37 isolates were *K. pneumoniae*. The collection comprised reference strains such as *K. pneumoniae* ATCC 700603, *E. coli* ATCC 25922, and *P. aeruginosa* ATCC 27853. Furthermore, the clinical isolates consisted of *A. baumannii*, *P. mirabilis*, two isolates of *K. oxytoca*, and two isolates of *K. ozaenae*. A comprehensive list of the bacterial isolates utilized in this study is presented in (S2 Table). The identity of the isolates was confirmed by Matrix-Assisted Laser Desorption Ionization Time of Flight (MALDI-TOF) mass Spectrometry (ZYBIO-EXS2600). Mueller-Hinton agar plate was used to perform disc diffusion testing using the 33rd edition of CLSI guidelines [36]. *K. pneumoniae* isolates that showed resistance to at least one antibiotic from three distinct classes of antibiotics were confirmed as MDR *K. pneumoniae* based on the Performance Standards for Antimicrobial Susceptibility Testing, 34^th^ Edition (CLSI M100) cut points [37]. The complete AST profile can be found in (S1 Fig)*. K. pneumoniae* ATCC®&700603 and *E. coli* ATCC®25922 obtained from the Ethiopian Public Health Institute (EPHI) were used for quality control in antimicrobial susceptibility testing. Two MDR *K. pneumoniae* hosts (TA-SP18, TA-SP92) were used for comparison of plaque and spot assay methods. The selected host isolates were grown in Tryptone Soy Broth (TSB) for 18 hours at 37°C for phage isolation.

### 2.3. Bacteriophage isolation and purification

#### 2.3.1. Spot assay.

The isolation host was cultured in TSB (OD_600_ = 0.4–0.5). Tryptone Soy Agar (TSA) was used to prepare the bottom agar (1.5% w/v), and Tryptone Soy Broth, and agarose 0.5% w/v were used to prepare the top (soft) agar according to a previous study [38] with little modification. The bottom agar was poured on 90 mm sized petri plates and dried in a working biological safety cabinet, while soft agar was kept at 45°C in a water bath. The plated petri plates were portioned into quadrants in such a way it leaves sufficient space between the spots, and labeled with the corresponding host, and samples to be spotted. Lawn was prepared in a 10 mL sterile test tube, by mixing 200 µL bacterial culture, 40 µL of (10 mM MgSO4), 40 µL of (mM CaCl2), and 3 mL 0.5% soft agar kept at 45°C. The mixture was then quickly poured onto the corresponding bottom agar. The soft agar was allowed to solidify for 5 minutes. The samples to be spotted were centrifuged at 10,000 rpm for 5 minutes before spotting. After centrifugation 20 µL from the supernatant of each sample was spotted on the lawn to the corresponding quadrant. The plates were incubated for 18 hours at 37°C, and examined for the presence of plaques or zone lysis, indicating the presence of lytic phages.

#### 2.3.2. Phage purification by streak plating.

The bacteriophages were further purified using streak plating [32]. For each sample that showed a positive result in the spot assay, a sterile inoculating loop was used to slightly touch the zone of lysis or plaque and streak it onto a fresh TSA plate in a zigzag pattern. Briefly using a sterile inoculating loop, the center of the plaque or clear zone observed on the spot assay plate was touched, and streaked in zigzag on the surface of the bottom agar in such a way that separated plaques can appear. After streaking, lawn was prepared as in the spot assay and slowly poured on the bottom agar from the end to the beginning of the streak so as not to erode phages from the high concentration areas as indicated in (Fig 1). The preparations were incubated for 18 hours at 37°C, and the next day assessed for the appearance of well-isolated single plaques. The step was repeated for three rounds of purification. Typical images obtained during streak plating-based phage purification can be found in (S2 Fig). Three steps-streaked plaque was picked using a sterile yellow pipette tip and resuspended in 1mL SM buffer using a 2 mL Eppendorf tube, slowly vortexed and incubated at 4°C for 6 hours. Then it was centrifuged at 10000 rpm for 10 minutes and filtered through a 0.22 µm sized syringe filter into a 1.5 Eppendorf tube and stored at 4°C for downstream characterization.

#### 2.3.3. Plaque assay.

A total of 124 plates were used to process simultaneously the two hosts on 62 samples with the spot assay method. Each bacterial host strain was grown in TSB until it reached an optical density of OD 600 of 0.132. The bottom and top (soft) agar was prepared as in spot assay. The bottom agar was poured onto 90 mm petri dishes and allowed to dry in a biological safety cabinet, while the soft agar was kept at 45°C in a water bath. To prepare the lawn, 200 µL of bacterial culture, 40 µL of 10 mM MgSO4, 40 µL of 2mM CaCl2, and 100 µL of the purified sample (which had been centrifuged for five minutes at 10,000 rpm before this assay) was mixed in a 10 mL sterile test tube. The preparations were incubated for 5 minutes at 37°C to allow the host to become infected, three milliliters of (0.5% w/v) soft agar, kept at 45°C, was added to the tube. The preparation was quickly poured onto the corresponding bottom agar and let to solidify at room temperature for 5 minutes. After 18 hours of incubation at 37°C, the plates were assessed for the appearance of plaques. Single plaque was picked using a sterile pipette tip and incubated for 24 hours at 4°C. The next day centrifuged at 10,000 rpm for 10 minutes, and the supernatant was filtered through a 0.22 µm syringe filter. The filtered samples were then prepared as 10-fold dilutions and used for the plaque assay. The step was repeated for three rounds of purification. The titer, host range, and stability at five different pH temperatures were assessed. The results were compared to the characteristics of phages isolated by spot assay isolation and streak plate purification methods on the corresponding hosts. Phage titration was conducted in the SM buffer using 10-fold dilutions. Plaque assay was conducted, and the bacteriophage titer in PFU/mL was calculated.

#### 2.3.4. Phage titer determination.

Phage suspensions were prepared in SM buffer at ten-fold serial dilutions according to the method described by Glonti et al [39]. The double-layer agar (DLA) method was then conducted using the host bacterial culture and each phage dilution. 1 mL of each phage dilution was mixed with 250 µL of the bacterium culture in the early exponential phase and 5 mL of 0.5% agar. The mixtures were homogenized and poured onto previously prepared plates with solid (bottom layer) agar. Finally, the plates were incubated overnight at 37°C, after which lysis plate counts were performed, and the number of plaque-forming units (PFU) for each dilution was established. The number of plaques per dilution was used to calculate the phage titration (PFU/mL).

**Table 1:** Summary of phages isolated against TASP18 and TASP92 using spot assay.

S.no	Screened samples	TASP18	TASP92	S.no	Screened samples	TASP18	TASP92	S.no	Screened samples	TASP18	TASP92
1	TTD	1	1	22	AKM	0	0	43	HRM	0	0
2	TTM	2	1	23	AKS	1	0	44	HRS	0	0
3	TAD	1	0	24	HGD	0	0	45	LAD	0	0
4	TAM	0	0	25	HGM	0	0	46	LAM	0	0
5	TBD	0	0	26	HGS	0	0	47	LAS	0	0
6	TBM	0	0	27	ETs01	0	0	48	JMD	0	0
7	TCD	0	0	28	ETs02	0	0	49	JMM	0	0
8	TCM	0	0	29	ETs03	0	0	50	JMS	0	0
9	TDD	0	0	30	GGD	1	0	51	SWD	0	0
10	TDM	0	0	31	GGM	0	1	52	SWM	0	0
11	TSL	0	0	32	GGS	0	0	53	SWS	0	0
12	ADD	0	1	33	KID	1	0	54	YHD	1	0
13	ADM	0	1	34	KIM	0	0	55	YHM	0	0
14	ADS	0	0	35	KIS	0	0	56	YHS	0	0
15	KFD	1	1	36	BYD	0	1	57	MHD	1	0
16	KFM	0	0	37	BYM	1	0	58	MHM	0	0
17	KFS	0	1	38	BYS	0	0	59	MHS	0	0
18	GWD	0	0	39	GKD	0	0	60	ZHD	0	0
19	GWM	0	0	40	GKM	1	1	61	ZHM	0	0
20	GWS	0	0	41	GKS	0	0	62	ZHS	1	0
21	AKD	0	0	42	HRD	0	0				

#### 2.3.5. Host range analysis.

All the phages isolated by both the spot assay and plaque assay were assessed for their host range using different bacterial isolates obtained from different clinical samples [40,41]. The assessment involved testing the isolated phages against 45 clinical isolates of bacterial strains listed in (Table S2). Briefly, 200 μL of each log-phase bacterial isolate was mixed with 3mL of soft agar and lawn onto a TSA bottom agar. Five microliters from the purified phage lysate were spotted. The preparation was incubated for 18 hours at 37°C. The appearance of the lysis zone on the spotted area was considered positive for phage activity.

**Table 2:** Summary of phages isolated against TASP18 and TASP92 using plaque assay.

S.no	Sample code	TASP18	TASP92	S.no	Sample code	TASP18	TASP92	S.no	Sample code	TASP18	TASP92
1	TTD	1	0	22	AKM	0	0	43	HRM	0	0
2	TTM	1	1	23	AKS	1	0	44	HRS	0	0
3	TAD	1	0	24	HGD	0	0	45	LAD	0	0
4	TA	0	0	25	HGM	0	0	46	LAM	0	0
5	TBD	0	0	26	HGS	0	0	47	LAS	0	0
6	TBM	0	0	27	ETs01	0	0	48	JMD	0	0
7	TCD	0	0	28	ETs02	0	0	49	JMM	0	0
8	TCM	0	0	29	ETs03	0	0	50	JMS	0	0
9	TDD	0	0	30	GGD	0	0	51	SWD	0	0
10	TDM	0	0	31	GGM	1	1	52	SWM	0	0
11	TSL	0	0	32	GGS	0	0	53	SWS	0	0
12	ADD	0	1	33	KID	0	0	54	YHD	1	0
13	ADM	0	1	34	KIM	0	0	55	YHM	0	0
14	ADS	0	0	35	KIS	0	0	56	YHS	0	0
15	KFD	0	0	36	BYD	0	1	57	MHD	1	0
16	KFM	0	0	37	BYM	1	0	58	MHM	0	0
17	KFS	0	1	38	BYS	0	0	59	MHS	0	0
18	GWD	0	0	39	GKD	0	0	60	ZHD	0	0
19	GWM	0	0	40	GKM	1	1	61	ZHM	0	0
20	GWS	0	0	41	GKS	0	0	62	ZHS	1	0
21	AKD	0	0	42	HRD	0	0				

#### 2.3.6. Determination of optimal multiplicity of infection.

To determine the optimal MOI, serial dilutions of the corresponding host *K. pneumoniae* grown to exponential phase (OD600 = 0.4–0.5) were mixed with phage at 1x10^8^ PFU/mL according to a method described by Fang et al [42]. Dilutions of 100, 10, 1, 0.1, 0.01, 0.001, and 0.0001 were added to the corresponding host bacteria. After four hours of incubation at 37°C the mixture was shaken and centrifuged at 10,000 rpm for five minutes. The supernatant was filtered through a 0.22 μm sized syringe filter. A plaque assay was conducted to determine phage titer. The experiment was conducted in triplicates. The bacteriophage’s titer was calculated and the MOI that gave the highest titer was regarded as the optimal one.

#### 2.3.7. Determination of Efficiency of Plating (EOP).

The efficiency of plating was determined to assess the infectivity of the isolated phages as previously described [43,44]. Bacteria strains that showed susceptibility during host range analysis were grown to (OD 600 = 0.4–0.5), and plaque assay was conducted on 10^−6^ − 10^−9^ fold diluted phage lysates. After conducting plaque assay in triplicates, the average number of plaques was counted. The efficiency of plating was determined by dividing the average PFU/mL of test bacteria to the average PFU/mL of host bacteria. EOP values were categorized as Low productive (0.001 < EOP < 0.1), medium productive 0.1 < EOP < 0.1), highly productive (EOP ≥ 0.5), and inefficient (≤0.001) [45].

#### 2.3.8. Stability under gradient temperatures and pH.

The stability of the eight selected phages stability at varying temperatures was investigated as previously described by Bai et al [46]. Specifically, the temperatures tested were room temperature (25°C), normal body temperature (37°C), and 40, 50, 60, and 70°C. Briefly, 300 microliters of the highest titer from each phage preparation were added to a 1.5 mL Eppendorf tube. The tubes were then incubated at the corresponding temperatures for an hour, followed by a slow cooling to room temperature. After incubation, the average phage titer at each temperature point was determined using a double-layer agar assay. The stability of phages under different pH conditions was also assessed in this study. The pH stability was conducted at five different pH values, specifically at pH 3, 5, 7, 9 and 11. To conduct this assessment, 500 µL of a phage suspension was transferred to a 5 mL TSB solution adjusted to the intended pH. The preparation was then incubated at 37°C for an hour. Three parallel experiments were conducted for each phage, and the average phage titer was determined using the double-layer agar technique.

#### 2.3.9. Single-step growth curve experiment.

Single-step growth curves were conducted to determine the burst size and latent period as previously described [47,48] with modifications. Briefly, the phages were mixed with their corresponding host (grown to OD_600_ = 0.4–0.5) at an MOI of 1 and incubated at 37°C for ten minutes to ensure maximum phage adsorption [49]. The preparation was then centrifuged at 10,000 rpm for one minute to remove non-adsorbed phages. The liquid part was discarded and the pellet was suspended in 5 mL fresh TSB. Plaque assay was conducted at 5, 15, 25, 35, 45, 55, 65 and 70 minutes. The average PFU/mL was calculated, and a single-step growth curve was plotted as described Peng et al [50].

### 2.4. Quality control

Quality control has been conducted in each experiment to ensure the accuracy and reliability of the experimental results. To assess the sterility of the media, bottom agar and soft agar without samples or host bacteria were used as a control. Contamination was considered if growth was observed on these plates. For filtrate control, filtered samples, expected to contain phages, were spotted onto the media prepared as in the media control, but without host bacteria. This was done to check for bacterial contamination in the filtrates. Filtrate contamination or inadequate filtration was indicated if growth was observed in this preparation but not on the media control. As host control the bottom agar was covered with a mixture of soft agar and host bacteria, without any spotted samples, to ensure that there was no intrinsic lysis. Experimental: The experimental setup involved mixing soft agar with the host bacteria and then preparing a lawn by pouring on the bottom agar, then spotting each sample to the corresponding quadrant as illustrated in (Fig 1). The presence of clear zones of lysis or plaques in this setup indicates the presence of lytic phages against the tested host.

### 2.5. Statistical analysis and data visualization

Statistical analysis and data visualization were performed using the R programming language, R version 4.4.1 (r-project.org), in R-studio. Data were visualized using the R built-in function and the ggplot2 package from the library tidyverse.

### 2.6. Ethical clearance

The study proposal was reviewed and approved by the Research Ethics Committee at the Institute of Biotechnology, Addis Ababa University (IoB/431/2016/2024). All methods were carried out as per the relevant ethical guidelines and regulations.

## 3. Results

### 3.1. Phage isolation

**Fig 2:**
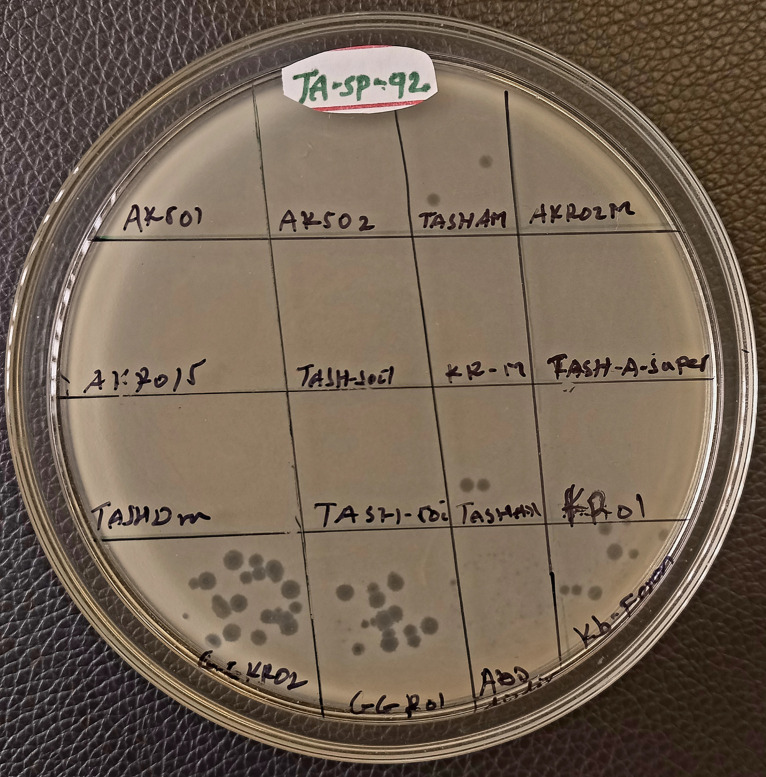
Representative images during spot isolation.

**Fig 3:**
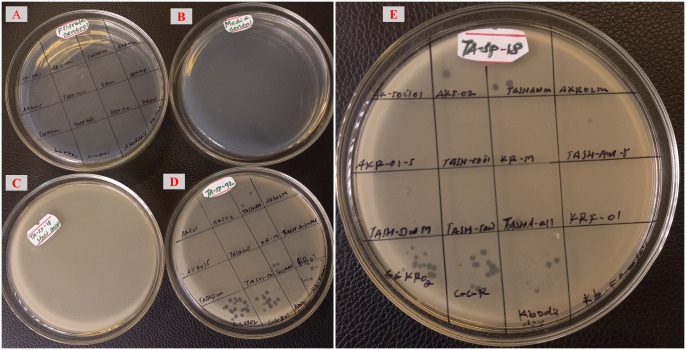
Illustrative images showing quality control results for experimental conditions. “**A**” **Filtrate control**: no growth was observed on the media spotted with sample filtrate prepared without host bacteria, indicating that the filtered samples were free from bacterial contamination confirming that the filtration process was sufficient and that any observed effects in experimental samples can be attributed to the phages. “**B**” **Media control**: no growth was observed on the bottom agar lawn with soft agar, confirming the sterility of the media used in the experiments. This suggests that the growth of bacteria in the experimental plate can be attributed to the host lawn. “**C**” **Host control**: there was no lysis in the host control, which was made up of soft agar with bottom agar and host bacteria validating the lack of lytic activity from any contaminants, making certain that any lysis seen in test samples is exclusively related to the samples being examined. “**D/E**” **Experimental results**: showing growth of host bacteria and presence of lytic phages against the tested host.

From the 62 samples screened, a total of 39 phages were isolated against the two MDR *K. pneumoniae* using the two methods: 22 phages were isolated by spot assay and 17 by plaque assay. Spot assay utilized 8 plates to screen 62 samples against the two hosts, while the plaque assay required 124 plates to screen the same number of samples. The phage recovery status from each sample for spot and plaque assays is shown in Tables 1 and 2, respectively. The representative images during spot isolation and quality control are shown in Fig 2 and 3, respectively.

**Table 3:** The titer of isolated phages.

S.no	Phages	Titer in PFU.mL^-1^	S.no	Phages	Titer in PFU.mL^-1^
1	TTDp-TASP18	2.4 x 10^5^	21	GKMs-TASP18	3.4 x 10^8^
2	TTMp-TASP18	1.6 x 10^5^	22	AKSs-TASP18	7.5 x 10^8^
3	TADp-TASP18	4.6 x 10^6^	23	KFDs-TASP18	4 x 10^5^
4	BYMp-TASP18	2.8 x 10^5^	24	GGDs-TASP18	3.6 x 10^6^
5	GGMp-TASP18	2.6 x 10^8^	25	KID s-TASP18	1.2 x 10^6^
6	AKSp-TASP18	8.2 x 10^9^	26	BYMs-TASP18	1.4 x 10^8^
7	GKMp-TASP18	7.2 x 10^5^	27	GKMs-TASP18	1.6 x 10^4^
8	YHDp-TASP18	2.4 x 10^6^	28	MHDs-TASP18	7.2 x 10^5^
9	ZHSp-TASP18	2.4 x 10^5^	29	YHDs-TASP18	2.5 x 10^7^
10	ADMp-TASP92	2.4 x 10^7^	30	ZHSs-TASP18	1.3 x 10^6^
11	KFSp-TASP92	2.4 x 10^4^	31	TTDs-TASP92	3.6 x 10^7^
12	KIDp-TASP92	5.6 x 10^8^	32	BYDs-TASP92	1.5 x 10^5^
13	TTMp-TASP92	1.3 x 10^9^	33	TTMs -TASP92	1.8 x 10^11^
14	GGMp-TASP92	2.4 x 10^6^	34	ADMs-TASP92	4.7 x 10^8^
15	BYDp-TASP92	5.6x 10^7^	35	ADDs-TASP92	6.3 x 10^6^
16	GKMp-TASP92	3.2 x 10^5^	36	GGMs-TASP92	3.8 x 10^7^
17	TTDs-TASP18	5.5 x 10^6^	37	KFDs-TASP92	4.7 x 10^4^
18	TTM1s-TASP18	6.4 x 10^4^	38	KFSs-TASP92	6.4 x 10^6^
19	TTM2s-TASP18	2.4 x 10^6^	39	GKMs-TASP92	7.4 x 10^5^
20	TADs-TASP18	1.3 x 10^7^			

**Table 4:** Host range analysis result of selected eight phages.

		Plaque-isolated phages				Spot-isolated Phages			
S.no	Bacteria	GGMp-TASP18	AKSp-TASP18	KIDp-TASP92	TTMp-TASP92	GKMs-TASP18	AKSs-TASP18	TTMs -TASP92	ADMs-TASP92
1	®ATCC700603	+	+	+	–	+	+	–	–
2	®ATCC25922	–	–	–	–	–	–	–	–
3	®ATCC27853	–	–	–	–	–	–	–	–
4	Ab14	–	–	–	–	–	–	–	–
5	Pm06	–	–	–	–	–	–	–	–
6	Kp05	–	–	–	–	+	–	–	–
7	Kp02	–	+	–	+	+	+	+	+
8	Kp03	–	–	+	–	–	–	–	–
9	Kp04	+	–	+	+	+	–	+	+
10	Kp01	–	–	–	+	–	–	+	–
11	Kox01	–	–	–	–	–	–	–	–
12	Kox02	–	–	–	+	–	–	+	–
13	Koz01	–	–	–	–	–	–	–	–
14	Koz02	–	–	–	–	–	–	–	–
15	TASP04	+	–	+	+	+	–	+	+
16	TASP06	+	+	+	+	+	+	+	+
17	TASP09	+	+	–	–	+	+	–	–
18	TASP17	–	+	–	–	–	+	–	+
19	TASP18	H	H	–	+	H	H	+	–
20	TASP22	–	+	+	+	+	+	+	+
21	TASP23	+	–	+	+	–	–	+	–
22	TASP28	+	–	+	–	–	–	–	–
23	TASP32	–	+	–	+	–	+	+	–
24	TASP37	–	+	–	+	–	+	+	–
25	TASP41	–	–	–	+	–	–	+	–
26	TASP45	–	+	+	+	+	+	+	+
27	TASP50	–	–	–	+	+	–	+	–
28	TASP54	–	–	–	–	+	–	–	–
29	TASP59	+	–	–	–	+	–	–	–
30	TASP65	–	–	–	+	+	–	+	–
31	TASP72	–	–	–	+	–	–	+	–
32	TASP76	+	+	+	–	+	+	–	–
33	TASP82	+	+	+	+	+	+	+	–
34	TASP-87	–	–	–	+	–	–	+	–
35	TASP88	+	–	+	+	+	–	+	–
36	TASP 92	–	–	H	H	–	–	H	H
37	TASP96	–	+	+	+	+	+	+	–
38	TASP101	+	–	–	–	–	–	–	–
39	TASP109	+	+	+	+	+	+	+	–
40	TASP110	–	–	–	+	–	–	+	–
41	TASP117	–	+	–	–	–	+	–	–
42	TASP118	+	–	+	+	+	–	+	–
43	TASP121	–	+	–	–	–	+	–	+
44	TASP126	–	+	–	+	+	+	+	–
45	TASP129	–	–	–	+	–	–	+	–
		15	17	16	25	20	17	25	8

**“H”** indicates the Isolation Host; **“+”** signifies the presence of a lysis zone; **“-”** indicates that no lysis zone was observed.

### 3.2. Phage titration

Titrations were conducted for all isolated phages. The highest titer, 1.8 x 10^11^, was observed on TTMs-TASP92, and the lowest titer, 1.6 x 10^4^, was observed on GKM s-TASP18 the complete phage titer results can be found in (Table 3). From this study phages that exhibited relatively higher titers and the ability to produce clear plaques on host bacteria lawn were selected for further characterization. Eight phages, namely, GGMp-TASP18, AKSp-TASP18, KIDp-TASP92, TTMp-TASP92, GKMs-TASP18, AKSs-TASP18, TTMs -TASP92, and ADMs-TASP92, were selected for downstream characterization including host range analysis, optimal multiplicity of infection, pH and temperature stability. This selection was judged mandatory to avoid the resource-intensive and tedious task of working with all phage isolates.

### 3.3. Analysis of host range

**Fig 4:**
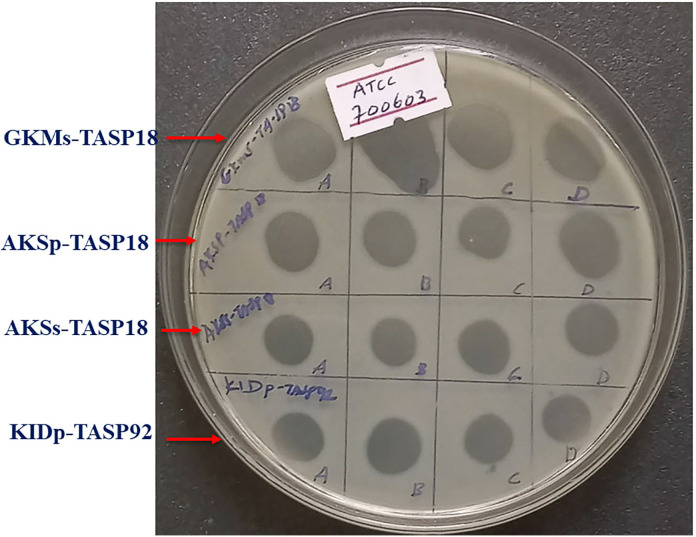
Illustrative images during host range analysis. “**A**” Lysis zones observed on *K. pneumoniae* ®ATCC 700603 during host range assessment by spot assay by four phages. The labeling written on the plate as A, B, C, and D indicates the replica spot of the corresponding phage.

Analysis of host range was conducted on the selected eight selected phages. To assess and determine their lysis spectrum, analysis was conducted using a panel of 45 bacterial strains indicated in (Table 2). Out of the eight phages tested against the 45 strains, including the isolation hosts, TTMs -TASP92 and ADMs-TASP92 were found to lyse 25 (45) 55.5% of the tested strains, indicating a broad lysis spectrum. However, ADMs-TASP92 lysed 8 out of 45 strains (17.7%). In this host range analysis, phage TTMs -TASP92 and ADMs-TASP92 were able to lyse *K. oxytoca*. Furthermore, the hosts TA-SP06, TA-SP82, and TA-SP109 were susceptible to all phages. On the other hand, the hosts TA-SP54, TA-SP101, Kp03, and Kp05 were only lysed by GKMs-TASP18, GGMp-TASP18, KIDp-TASP92, and GKMs-TASP18, respectively. In contrast, none of the phages showed any lytic activity against *E. coli* ®ATCC25922, *P. aeruginosa* ®ATCC27853, *A. baumannii* (Ab14), *P. mirabilis* (Pm06), *K. oxytoca* (Kox01), and all the tested *K. ozaenae* strains. A comprehensive overview of the lysis results for each phage and illustrative images taken during host range analysis can be found in Table 4 and Fig 4, respectively.

### 3.4. Efficiency of plating (EOP)

**Fig 5:**
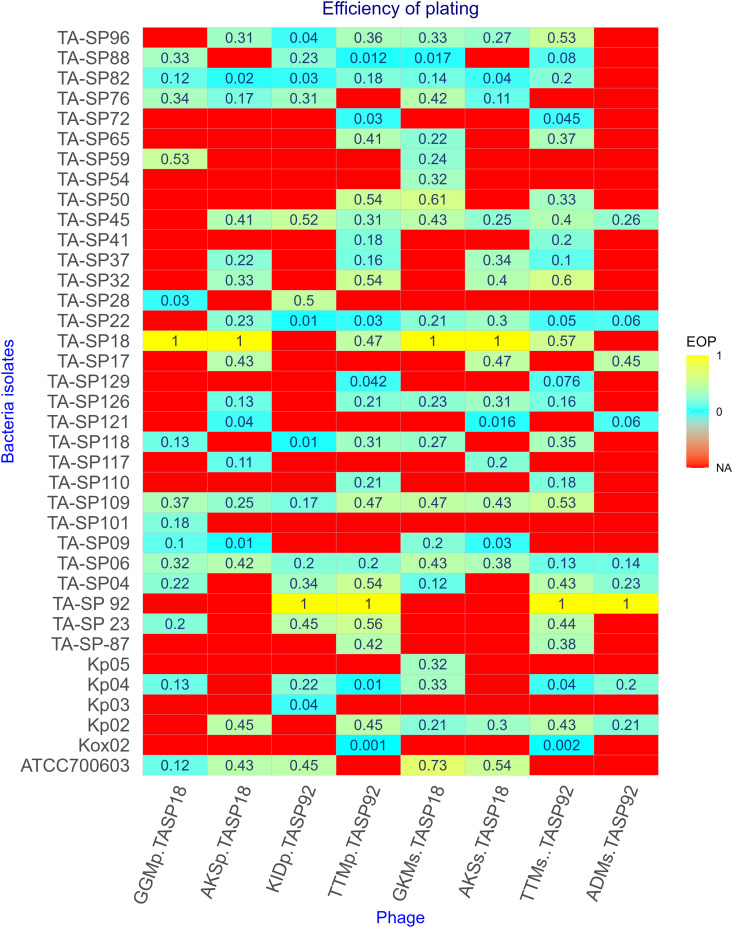
Phage efficiency against various bacterial strains. EOP values were categorized as Low productive (0.001 < **EOP** < 0.1), medium productive 0.1 < **EOP** < 0.1), highly productive (**EOP **≥ 0.5), and inefficient (≤0.001) a value 1 indicates isolation host. The red color in the heat map indicates “NA” not applicable; indicating the phage did not lyse the corresponding bacteria.

**Fig 6:**
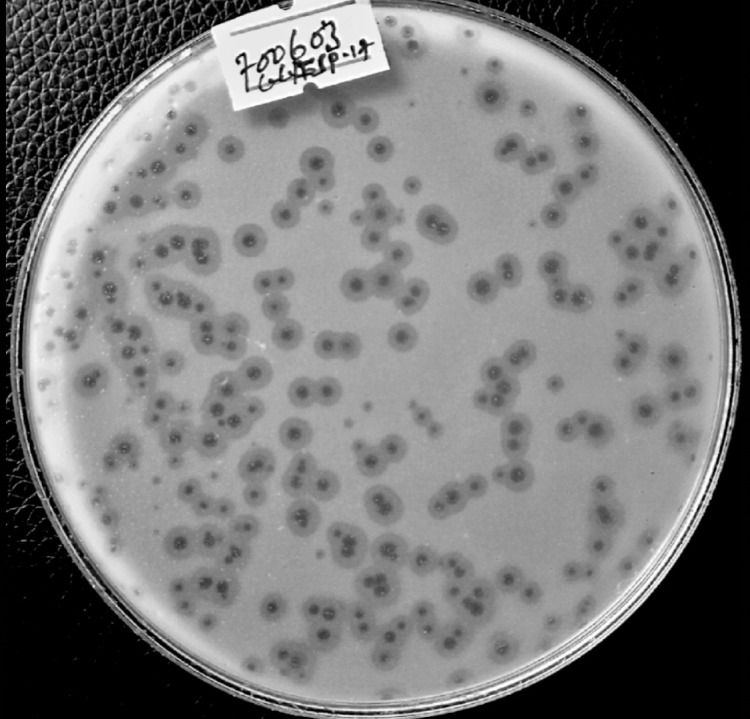
Illustrative images during EOP. Plaque of phage GKMs-TASP18 on *K. pneumoniae* ®ATCC 700603 during EOP. Optimum Multiplicity of infection (MOI) of selected phages.

The infectivity of the selected phages was assessed using their EOP. Thirty-seven bacterial strains that showed susceptibility in the spot test during host range analysis were subjected to the EOP test. Among the tested phages, phage GKMs-TASP18 and TTMs-TASP92 exhibited a high EOP on *K. pneumoniae* ®ATCC 700603 and TA-SP18, respectively. Phages TTMs-TASP92 and TTMp-TASP92 were inefficient on *K. oxytoca* (Kox02). The complete EOP profile and illustrative images during EOP are shown in Figs 5 and 6, respectively.

The investigation of the optimal multiplicity of infection (MOI) for the eight selected phages yielded values between 0.1 and 10. The phages TTMp-TASP92 and TTMs-TASP92 exhibited the highest average titers at an MOI of 1, measuring 2 x 10^9 PFU/mL and 1.6 x 10^9 PFU/mL, respectively. Both AKSp-TASP18 and AKSs-TASP18 demonstrated significant titers of 8.8 x 10^8 PFU/mL and 8 x 10^8 PFU/mL at a MOI of 0.1, suggesting that even lower phage concentrations can still produce sizable viral loads. The comprehensive MOI value and the corresponding highest titer observed can be found in (Table 5).

**Table 5:** Optimal multiplicity of infection of eight phages.

S.no	Phage	MOI	Highest average titer
1	AKSs-TASP18	0.1	8 x 10^8^
2	AKSp-TASP18	0.1	8.8 x 10^9^
3	KIDp-TASP92	0.1	6 x 10^8^
4	TTMp-TASP92	1	2 x 10^9^
5	ADMs-TASP92	10	5 x 10^8^
6	GKMs-TASP18	10	2.9 x 10^8^
7	TTMs -TASP92	1	8.6 x 10^10^
8	GGMp-TASP18	0.1	2.2 x 10^8^

### 3.5. Single-step curve experiment

**Fig 7:**
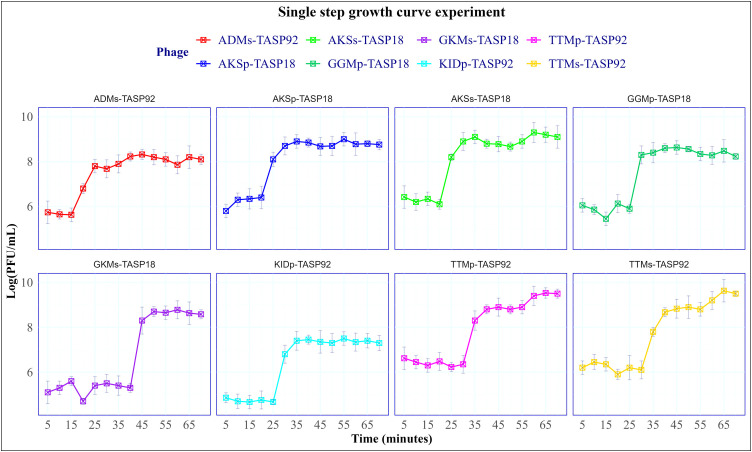
Single-step growth curve experiment of isolated phages. The data points represent the average Log (PFU/mL) at each incubation time, with standard deviation (SD) indicated by vertical lines on the Y-axis. The data were analyzed using a one-way ANOVA followed by Tukey’s HSD test (***P < 0.0059).

Single-step growth curve experiments were conducted on the eight selected phages, which were enriched using two methods: spot assay and plaque assay on two hosts. The experiment aimed to assess the growth characteristics of each phage. The findings showed that the phages exhibited different patterns regarding their latent periods and burst sizes. The latent periods ranged from 15 minutes for ADMs-TASP92–40 minutes for GKMs-TASP18, indicating variations in their replication cycles. Additionally, the burst sizes varied among the phages, with TTMp-TASP92 showing the highest burst size of 310, and KIDp-TASP92 the lowest burst size of 76. AKSp-TASP18 and AKSs-TASP18 both exhibited a latent period of 20 minutes and burst sizes of 118 and 126, respectively. TTMp-TASP92 and TTMs-TASP92 shared the same latent period of 30 minutes, and burst size of 310 and 298 viral particles, respectively. The results of single-step growth curve experiments are presented graphically in Fig 7.

### 3.6. Phage stability across varying pH values

**Fig 8:**
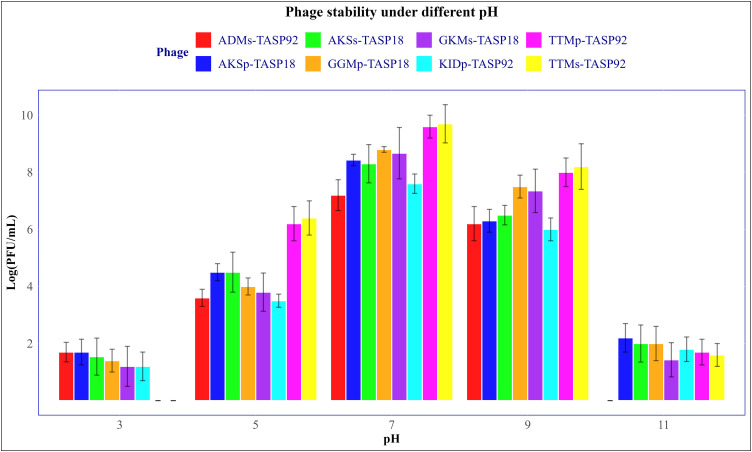
Phage stability under different pH gradients. The bar represents the titer of eight phages at five different pH values 3, 5, 7, 9, and 11. The data point indicates the mean titer in PFU/mL. SD is indicated using vertical lines on the Y-axis. The data were analyzed using a one-way ANOVA, followed by Tukey’s HSD test (**P < 0.003).

The stability of the eight selected phages was assessed at five different pH values: 3, 5, 7, 9, and 11. The results are presented as mean ± standard deviation (SD) in Fig 8. All tested phages exhibited higher lytic activity at pH values between 5 and 9. The highest phage activity was observed at pH 7. Two phages, TTMp-TASP92 and TTMs-TASP92 showed no lytic activity. Conversely, phage ADMs-TASP92 showed no lytic activity at pH 11.

### 3.7. Phage stability under different temperatures

**Fig 9:**
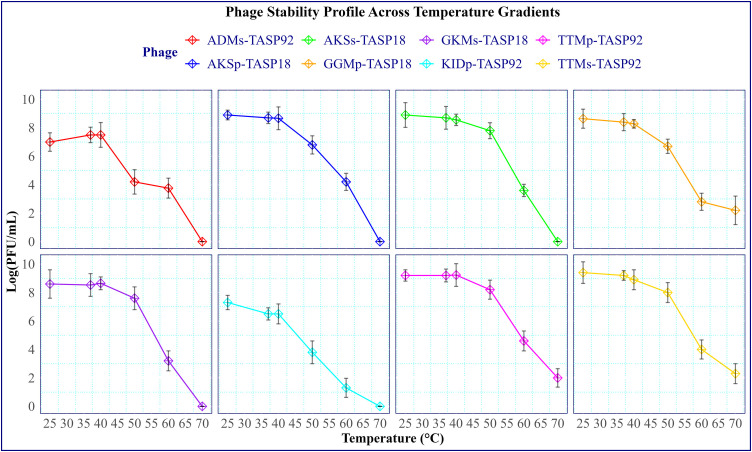
Phage viability across a temperature gradient. The data points represent the average titer. Standard deviation is indicated using vertical lines on the Y-axis. The data were analyzed using a one-way ANOVA, followed by Tukey’s post hoc test (**P < 0.04).

Phage stability across different temperatures was assessed at five temperatures: 25°C (room temperature), 37°C (normal body temperature), and 40, 50, 60, and 70°C. The viability of examined phages was assessed across the temperature gradients as graphically illustrated in Fig 9. Despite variations in titer, all phages remained stable and viable within the temperature range of 25°C to 60°C. Although their titers decreased by approximately 90% compared to those at 25°C, the three phages-GGMp-TASP18, TTMs-TASP92, and TTMs-TASP92-were able to survive at 70°C. Five phages’ ADMs-TASP92, AKSs-TASP18, AKSp-TASP18, and GKMs-TASP18 showed no lytic activity at 70°C.

## 4. Discussion

The increasing prevalence of antibiotic-resistant bacterial pathogens makes isolating bacteriophages targeting *K. pneumoniae* more important than ever. Environmental reservoirs, including hospital surroundings like wastewater, sinks, and surfaces, play a key role in the persistence and spread of multidrug-resistant (MDR) bacteria such as *K. pneumoniae* [51]. These environments harbor diverse, often antibiotic-resistant bacterial populations that can colonize vulnerable patients and contribute to healthcare-associated infections [52]. Importantly, the diverse bacteriophages found in these samples enable isolation of phages that specifically target pathogenic MDR bacteria. Such phages hold promise as alternative or adjunct therapies to antibiotics, especially against resistant strains, offering a valuable strategy to combat infections and reduce the burden of MDR pathogens in hospital settings.

The presence of a diverse range of bacteriophages found in environmental samples allows for the isolation of phages that are specific to the pathogenic bacteria, making them suitable for phage therapy applications. In this study, we collected samples from various environmental sources, including wastewater, sewage effluents, and soil, to isolate lytic phages against MDR *K. pneumoniae*. Conventional phage isolation procedures involve filtering environmental samples like wastewater to remove contaminants, and then combining the filtrate with host bacteria to encourage phage amplification. However, this method is frequently criticized for requiring a lot of resources and having a poor success rate when it comes to phage isolation [15,53].

Spot tests were used in this investigation, and the results showed clear lysis patterns, suggesting strong phage activity against the target *K. pneumoniae* strains. This finding shows that the environmental areas tested may have a rich diversity of phages capable of infecting *K. pneumoniae,* which is encouraging for future phage therapy applications [54]. Environmental sources, including hospital sewage and soil, serve as natural reservoirs of diverse bacteriophages that can infect pathogenic bacteria circulating in clinical settings. Phages isolated from these environments have direct clinical relevance because hospital sewage often contains multidrug-resistant bacterial strains shed by patients, making it a rich source of phages capable of targeting clinically important pathogens. By isolating phages from such environments, we harness naturally occurring viral predators that have evolved to infect and lyse bacteria prevalent in healthcare settings [55].

Although it is lengthy and tedious, it is crucial to recognize that enrichment methods continue to be the gold standard for phage separation when phage concentrations are expected to be low, as they increase the possibility of isolating phages from samples with sparse phage populations. The conventional plaque assay procedure usually entails several steps: selecting plaques, suspending them in a buffer, purifying the lysate the next day, carrying out serial dilutions, and testing each dilution on a single host. These are the differences between our purification method and standard purification assays. It can take more than a week or more to isolate and purify a single phage using the plaque assay, and resource consumption increases exponentially with larger sample sizes.

The generalized approach of the phage isolation technique adapted by van Twest and Kropinski [56] still serves as the foundation. Despite poor isolation success rates, prior research indicates that direct spotting techniques have resulted in observable plaques on *K. pneumoniae* strains [57]. Phages are isolated from waste-water samples by filtering the contaminants. The filtrate is mixed with host bacteria to enable host cell infection and phage amplification. However, this is labor-intensive and time-consuming. The approach not only simplifies the purification of isolated phages but also reduces the resource and time consumed during sample processing. By increasing the concentration of the samples, the study implemented an alternative approach that did not require enrichment and yielded good results. This method is efficient as enrichment is frequently a laborious procedure. Instead, the study achieved successful results by simplifying the sample purification process by increasing the concentration of the samples. By centrifuging the filtered materials after filtering a large volume, the concentration of the samples was increased. This study successfully isolated and purified phages using spot assay and streak plating, respectively.

The study results indicate a difference in the rate of phage isolation between the spot assay and the plaque assay. Using the spot assay, 22 phages were isolated from 62 samples using only 8 plates, while the plaque assay yielded 17 phages from the same number of samples but required 124 plates, consuming 16-fold resource compared to plaque assay. The variation can be attributed to various factors associated with the isolation method. Although the spot assay is not commonly used for phage isolation, it is recognized for its sensitivity in detecting phage activity [28]. In this assay, the samples are directly applied onto a bacterial lawn, enabling the observation of phage lytic activity. The spot assay is capable of producing distinct areas of lysis even with relatively low phage concentrations infection at the spot. Conversely, the plaque assay relies on the formation of discrete plaques. Some phages cannot form plaques on productive hosts because their diffusion in agar is limited or their productivity is low.

In this study, the majority of the isolated phages were obtained from homogenized samples rather than discrete ones. This suggests that the act of mixing or homogenizing samples may enhance the representativeness of the sample, thereby increasing the likelihood of detecting phages that may have otherwise been overlooked during sampling. Studies reveal that homogenization not only helps produce a more homogeneous sample but also increases the likelihood of detecting a greater diversity of phages present in environmental or biological samples [58]. In addition, there is a significant difference in the resources required for the two assays. The spot assay yields a higher number of phages compared to the plaque assay, which can be attributed to the spot assay’s sensitivity [59]. All of these factors emphasize the importance of selecting the appropriate method for phage isolation and purification [60,61].

Purifying bacteriophages using the streak plating method is not only an efficient approach regarding resource- and time-efficient, especially when dealing with temperature-sensitive bacteria and phages. This approach has various benefits and solves the problems with previous studies using streaking plating on soft agar preparation to see phage activity. The novelty of this method lies in its ability to effectively purify phages while maintaining the viability of temperature-sensitive bacteria and phages. By streaking before pouring and carefully controlling temperatures, this method mitigates risks associated with thermal shock and the effect of media scratches on results. The temperature can be maintained at an appropriate level for the particular bacteria and phages by streaking the phage plaque before adding the soft agar. This way, there is no risk of the soft agar solidifying right away or the need for extremely high temperatures that might be harmful to the organisms. The use of soft agar at temperatures higher than 50°C can be hazardous to thermo sensitive bacteria and phages, perhaps resulting in phage inactivation or cell death. Previous studies have indicated that higher temperatures above 45°C can cause phage inactivation and cell death [62–64]. The studies have also explored the significance of temperature in phage survival and activity.

On the other hand, rapid solidification can happen if the temperature is kept at 40–45°C during pouring, which could cause media scratches [62,65]. To reduce the possibility of media scratches, which might produce false-positive results, the soft agar is gradually poured in and streaked under supervision. After streaking, a soft agar lawn is made and applied to the bottom agar starting at the end of the streak and working its way towards the beginning. By using this method, erosion during pouring is prevented and disturbance to phage concentration zones is minimized. Even at higher temperatures, the integrity and viability of the phage and the bacteria can be preserved by streaking the phage plaque on the bottom agar before adding the bacterial host and soft agar lawn. To prevent the phages from building up in any one place and to promote the creation of well-separated plaques, it is important to pour the soft agar from the end of the streak toward its beginning.

In the phenotypic characterization of the selected eight phages, AKSp-TASP18 and AKSs-TASP18 showed similar characteristics. A similar observation is noted between TTMp-TASP92 and TTMs-TASP92. These phages, despite being isolated using different methods, showed distinct latent periods: 20 minutes for AKSp-TASP18 and AKSs-TASP18, and 30 minutes for TTMp-TASP92 and TTMs-TASP92. Furthermore, their burst sizes were comparable, with 118 and 126 for the first pair, and 310 and 298 for the second. Another similarity is in their host range: the first pair lysed 37.7%, while the broader host range of 55.5% observed for the second pair highlights the effectiveness of these phages against a wide range of MDR *K. pneumoniae*, emphasizing the importance of species-specificity and broad host range in phage therapy efficacy. These findings were consistent with previous work that has studied phage efficacy against *K. pneumoniae*; certain bacteriophages could effectively lyse approximately 63% of Klebsiella strains [66], which aligns with the host range results observed in this study. The ability of the phages to target a wider array of strains could enhance their utility in clinical settings, especially given the rising prevalence of antibiotic resistance. This suggests that isolated phages could be potential candidates for phage therapy.

The host range analysis of isolated phages against *K. pneumoniae* and other species provides valuable insights into their potential application in therapeutic settings, especially in the face of rising antibiotic resistance. In this study, eight phages were tested against a panel of 45 bacterial strains, including 37 *K. pneumoniae* strains, *K. oxytoca*, *K. ozaenae*, *Acinetobacter baumannii, E. coli, Pseudomonas aeruginosa, and Proteus mirabilis*. The results revealed a varying lytic spectrum among the phages analyzed. TTMs-TASP92 and ADMs-TASP92 showed a broader lysis spectrum, with the ability to lyse 25 out of 45 strains (55.5%). The fact that TTMs-TASP92 and ADMs-TASP92 can lyse *K. oxytoca* suggests their potential use in treating infections caused by this species. On the other hand, phage ADMs-TASP92 exhibited a narrow lysis spectrum, only lysing 17.7% of the tested strains. This specificity may indicate that ADMs-TASP92 is a more specialized phage that infects a smaller number of bacterial hosts. The specificity of phages towards their bacterial hosts is crucial, as it can be advantageous for precision treatment of specific bacterial strains.

The host range analysis of isolated phages against *K. pneumoniae*, and other species highlights significant insights into the potential application of these phages in therapeutic settings, particularly in the face of rising antibiotic resistance. In this study, eight phages were tested against a panel of 45 bacterial strains, including 37 *K. pneumoniae* strains, *K. oxytoca*, *K. ozaenae*, *A. baumannii*, *E. coli, P. aeruginosa, and Proteus mirabilis.* The results showed a varying lytic spectrum. Among host range analyzed phages, TTMs-TASP92 and ADMs-TASP92 exhibited a broader lysis spectrum, successfully lysing 25 out of 45 strains (55.5%).

The capability of TTMs-TASP92 and ADMs-TASP92 to lyse *K. oxytoca* suggests a potential for these phages to be used in treating infections caused by this species. On the other hand, phage ADMs-TASP92 showed a narrow lysis spectrum, effectively lysing only 17.7% of the tested strains. This specificity could suggest that the phage is specialized to infect a limited number of bacterial hosts. The specificity of phages towards their bacterial hosts is crucial. This might be advantageous in cases where precision treatment of limited bacterial strains is planned [67].

The analysis of the single-step growth curve revealed variability in phage replication, specifically in terms of latent durations and burst sizes. The latent period, which measures the time it takes for the phages to complete their replication cycle, varied from 15 minutes for ADMs-TASP92–40 minutes for GKMs-TASP18. This variation aligns with previous research by Zhao et al [41], which demonstrated that different phages can have significantly different replication times due to their interactions with host cells and the effectiveness of their infection mechanisms. ADMs-TASP92, with its shorter latent time, suggests a faster replication cycle that could be beneficial in situations requiring rapid proliferation. Additionally, there were significant differences in the burst size of each phage, indicating the number of additional phage particles released per infected cell. TTMp-TASP92 exhibited the largest burst size of 310 phage particles. In contrast, KIDp-TASP92 had the lowest, producing only 76 virions. This characteristic can be beneficial in situations where a slow but persistent infection is desired.

The analysis of selected phages for pH stability showed that all tested phages were most effective at a pH close to neutral. However, their effectiveness decreased at higher and lower pH values. These findings are crucial for determining how to use these phages in environmental and medical settings. Although TTMp-TASP92 and TTMs-TASP92 had the highest titers between pH 5 and 9, they could not form plaques after being exposed to pH 3 for an hour. On the other hand, phage ADMs-TASP92 was unable to form plaques at pH 12. The pH stability results for the tested phages, which showed optimal activity near neutral pH and decreased activity at more extreme pH values, aligns with previous studies.

Studies have indicated the significance of physiologic factors, including pH, on the stability and infectivity of phages. For example, a study on *K. pneumoniae phage* vB_kpnM_17–11 indicated that phage infectivity is highly dependent on physiologic conditions, including pH, with optimal activity between pH 4.8 and 8. This indicates that vB_kpnM_17–11 may lose viability or become inactive at pH levels outside its optimal range, which has implications for its application, such as limitations in oral administration due to the acidic gastric environment. Research on enhancing the stability of phages indicated that phages generally encounter instability and loss of activity when exposed to sudden environmental changes, including pH variations. This emphasizes the need for optimized storage and application conditions that can stabilize phages under different conditions, which is crucial for their utilization in various fields [68]. Another study evaluating phage adsorption to *Salmonella Typhimurium* indicated that phage P22 could not lyse the host at acidic pH but remained stable above pH 4 [69].

Based on the results from this study, TTMp-TASP92 and TTMs-TASP92 failed to form plaques after treatment at pH 3, and phage ADMs-TASP92 failed to form plaques at pH 12, consistent with previous studies [64,65]. These findings highlight the importance of considering phage pH stability when developing phage-based applications. Extreme pH conditions can significantly impair phage efficacy; thus, ensuring their maximum effectiveness across varying conditions is crucial. By considering the pH stability, steps can be taken to improve their stability and effectiveness. For instance, their formulation can be modified to increase the range of pH values they can tolerate, or they can be used in settings where pH levels are controlled.

The pH stability profile of these phages is particularly important in environmental and medical applications. The isolated phages are most effective at pH levels from 5 to 9 and are well-suited for phage therapy, which typically involves introducing phages into biological fluids or tissues. This aligns with the normal pH of human tissues and bodily fluids, increasing the chances of successful therapy. However, without further formulation or stabilization, these phages may be less effective in conditions with extreme pH variations- such as highly acidic or alkaline environments- due to their lack of activity at extreme pH levels.

Phage stability across different temperatures was investigated at 25°C, 37°C, 40°C, 50°C, 60°C, and 70°C. The phages showed stability and maintained their viability within temperatures ranging from 25°C to 60°C. At 60°C, although titers were significantly reduced, all phages survived the exposure. However, at 70°C, only three phages survived, with titers reduced by approximately 90% compared to those at 25°C. The other five tested phages showed no lytic activity at 70°C. These results indicate the stability of phages across different temperatures, particularly in the context of environmental and therapeutic applications. Phage stability was maintained up to 60°C, indicating their viability and effectiveness at moderately high temperatures. This aligns with previous studies that illustrate the importance of environmental conditions on phage viability [46,70–72].

Phage stability between 25°C and 60°C provides insight into their ability to withstand mild heat conditions. This temperature range encompasses normal physiological and ambient temperatures. Previous research by Kering et al [73] indicated that many phages remain stable and active at moderate temperatures. These phages maintained their viability and displayed effective lytic activity within this temperature range. Furthermore, it is important to note that these phages can survive and remain active even at temperatures as high as 60°C [73]. This suggests that they may have practical applications in scenarios where elevated temperatures are encountered, such as certain industrial processes or settings where heat is a concern.

Although phage titers decreased significantly at 60°C, the survival of all phages indicates they possess some degree of thermal resilience. This reduction in titer is likely due to the denaturation of phage proteins and possible disruption of phage structure, as discussed in previous literature [74]. Nonetheless, their survival indicates a relatively high heat tolerance compared to many other phages, which can be advantageous in applications involving moderate temperatures.

## 5. Strengths of the study

The strengths of this research include the use of an efficient combined spot assay and streak plate method, which allows for the screening of large numbers of samples with limited resources by significantly reducing time and labor compared to traditional plaque assays. The isolated phages were thoroughly characterized, including assessment of their latent periods, and exhibited broad-spectrum lytic activity against MDR *K. pneumoniae*, as well as stability across a wide pH range and temperatures up to 60°C. This efficient isolation technique facilitates the recovery of diverse and potent phages for further evaluation of their therapeutic potential.

## 6. Limitations of the study

Not all isolated phages were characterized, which restricts understanding of phage diversity and potential applications. Molecular characterization was not conducted, which limits insights into the genetic makeup and mechanisms of action of the phages. These limitations highlight the need for further research to improve characterization methods and expand resources for more comprehensive studies on phage therapy against antibiotic-resistant bacteria.

## 7. Conclusion and recommendations

The combined spot assay and streak plating method proved to be an efficient method for phage isolation, producing results comparable to traditional plaque assays while reducing time and resources. Characterization of the isolated phages revealed a diverse lytic spectrum, with some exhibiting broad host ranges against various *K. pneumoniae* isolates and related species. Notably, several phages displayed significant lytic activity. Analysis of growth characteristics showed variability in latent periods and burst sizes among the phages, indicating different replication dynamics that could be advantageous for therapeutic applications. Stability studies demonstrated that the isolated phages maintained effective lytic activity at pH levels between 5 and 9 and temperatures up to 60°C, underscoring their potential for use in diverse environmental and clinical settings. These findings highlight the need for further genomic characterization of the isolated phages to confirm their strictly lytic nature and verify the absence of undesirable genes, thereby enhancing their suitability for therapeutic use.

## Supporting information

S1 Fig AST profile of *K. pneumoniae* isolates used in phage isolation (DOCX)

S2 Fig Illustrative images during streak plate based phage purification. (DOCX)

S1 Table Summary of collected samples for bacteriophage isolation. (DOCX)

S2 Table List of bacteria isolates used in this study. (DOCX)

## Abbreviations

AMR: Antimicrobial Resistance
ATCC: American Type Culture Collection
CDC: Centers for Disease Control and Prevention
EOP: Efficiency of Plating
MDR: Multi-drug Resistant
MOI: Multiplicity of Infection
OD: Optical Density
TSA: Tryptone Soy Agar
TSB: Tryptone Soy Broth.
