# Supplementary material for: Isolation, purification, and phenotypic characterization of virulent Klebsiella pneumoniae phages from environmental samples in Addis Ababa, Ethiopia: A synergistic approach combining spot assay and streak plating
Source: PLoS One. 2025 Sep 24;20(9):e0331955. doi: 10.1371/journal.pone.0331955 (PMC12459788; doi:10.1371/journal.pone.0331955)
Supplement: S1 Fig — (DOCX) [file pone.0331955.s001.docx]

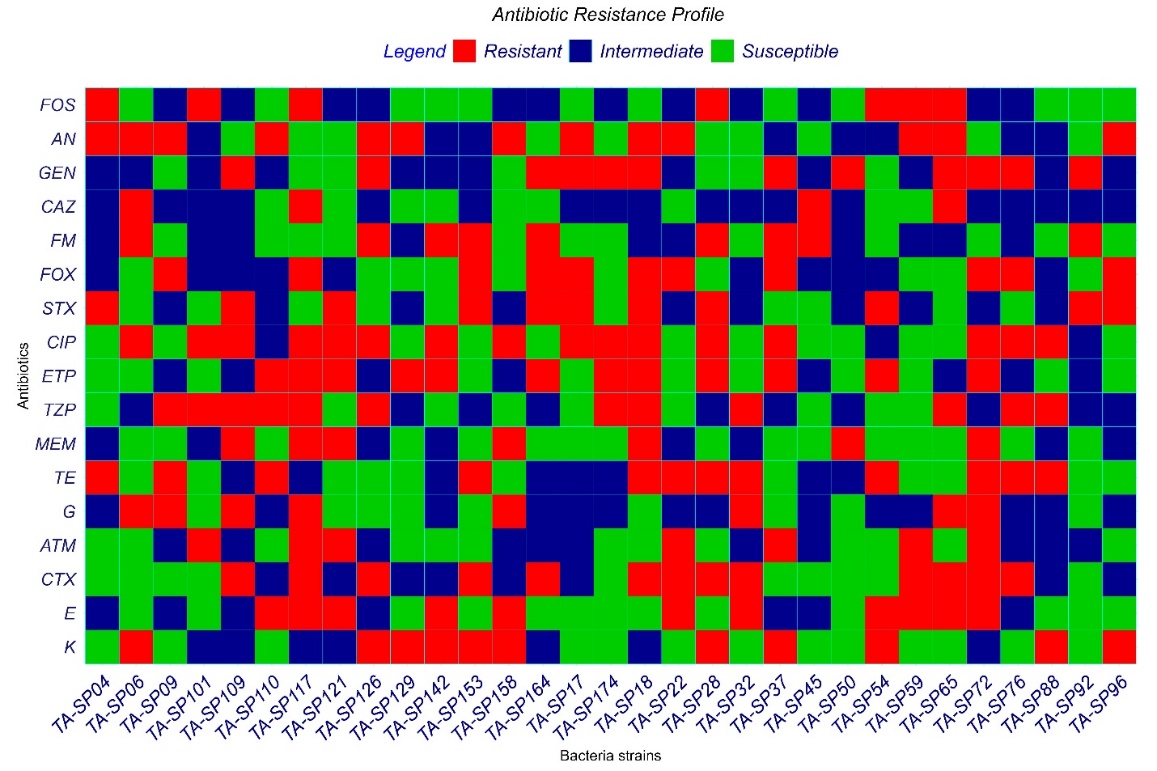


**S1 Fig.**  AST profile of *K. pneumoniae* isolates used in phage isolation. “**FOS**” Fosfomycin, “**AN**” Amikacin, “**GEN**” Gentamycin, “**CAZ**” Ceftazidime, “**FM**” Nitrofurantoin, “**FOX**” Cefoxitin, “**STX**” Sulfamethoxazole/trimethoprim, “**CIP**” Ciprofloxacin, “**ETP**” Ertapenem, “**TZP**” Piperacillin/tazobactam, “**MEM**” Meropenem, “**TE**” Tetracycline, “**G**” Sulfisoxaxole, “**ATM**” Aztronam, “**CTX**” Cefotaxime, “**E**” erythromycin, “**K**” Kanamycin.
