# Supplementary material for: Isolation, purification, and phenotypic characterization of virulent Klebsiella pneumoniae phages from environmental samples in Addis Ababa, Ethiopia: A synergistic approach combining spot assay and streak plating
Source: PLoS One. 2025 Sep 24;20(9):e0331955. doi: 10.1371/journal.pone.0331955 (PMC12459788; doi:10.1371/journal.pone.0331955)
Supplement: S2 Fig — (DOCX) [file pone.0331955.s002.docx]

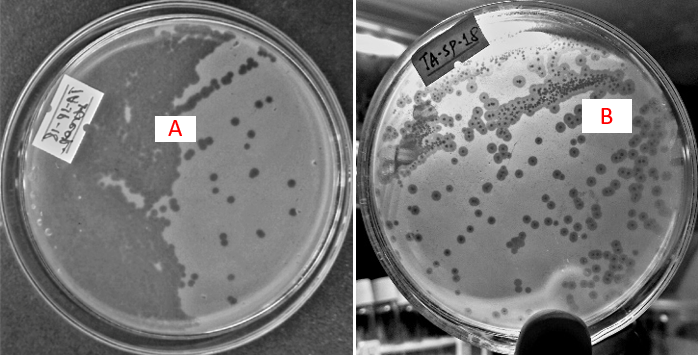


**S2 Fig**. Illustrative images during streak plate phage purification “A” Second round streak plate of phage AKSs-TA-SP18 on the host TA-SP18, “B” third round streak plate of phage GKMs-TASP18 on the host TA-SP18.
