# Supplementary material for: Isolation, purification, and phenotypic characterization of virulent Klebsiella pneumoniae phages from environmental samples in Addis Ababa, Ethiopia: A synergistic approach combining spot assay and streak plating
Source: PLoS One. 2025 Sep 24;20(9):e0331955. doi: 10.1371/journal.pone.0331955 (PMC12459788; doi:10.1371/journal.pone.0331955)
Supplement: S1 Table — (DOCX) [file pone.0331955.s003.docx]

**Table 1. Summary of collected samples for bacteriophage isolation**

| **S.no** | **Sampling site** | **Collected sample type** | **Sample Code** |
| --- | --- | --- | --- |
| 1 | Tikur Anbessa Specialized Hospital (TASH) | TASH combined (total) sewage tank discrete (TTD) | TTD |
|  |  | TASH combined (total) sewage tank mixed (TTM) | TTM |
|  |  | TASH A ward sewage discrete (TAD) | TAD |
|  |  | TASH A ward sewage mixed (TAM) | TAM |
|  |  | TASH B ward sewage discrete (TBD) | TBD |
|  |  | TASH B ward sewage mixed (TBM) | TBM |
|  |  | TASH C ward sewage discrete (TCD) | TCD |
|  |  | TASH C ward sewage mixed (TCM) | TCM |
|  |  | TASH D ward sewage discrete (TDD) | TDD |
|  |  | TASH D ward sewage mixed (TDM) | TDM |
|  |  | Soil from TASH (TSL) | TSL |
| 2 | Abo Dildiy Wastewater (ADW) | Abo Dildiy River discrete (ADD) | ADD |
|  |  | Abo Dildiy River mixed (ADM) | ADM |
|  |  | Soil near Abo Dildiy River (ADS) | ADS |
| 3 | Kebena River (French Embassy area) (KRF) | Kebena River (French Embassy area) discrete (KFD) | KFD |
|  |  | Kebena River (French Embassy area) mixed (KFM) | KFM |
|  |  | Soil near Kebena River (French Embassy area) (KFS) | KFS |
| 4 | Great Akaki River (AKR) | Akaki River discrete (AKD) | AKD |
|  |  | Akaki River mixed (AKM) | AKM |
|  |  | Soil near Akaki river (AKS) | AKS |
| 5 | Habte Georgis River (HGR) | Habte Georgis River discrete (HGD) | HGD |
|  |  | Habte Georgis River mixed (HGM) | HGM |
|  |  | Soil near Habte Georgis River (HGS) | HGS |
| 6 | Entoto 01 (ET01) | Soil from Entoto 01 (ETS01) | ETS01 |
| 7 | Entoto 02(ET02) | Soil from Entoto 02 (ETS02) | ETS02 |
| 8 | Entoto 03 (ET03) | Soil from Entoto 03 (ETS03) | ETS03 |
| 9 | Gedelgibu River (GGR) | Gedelgibu River Discrete (GGD) | GGD |
|  |  | Gedelgibu River Mixed (GGM) | GGM |
|  |  | Soil near Gedelgibu River (GGS) | GGS |
| 10 | Kebena River (Embassy of Italy area) (KRI) | Kebena River (Embassy of Italy) area discrete (KID) | KID |
|  |  | Kebena River (Embassy of Italy) area mixed (KIM) | KIM |
|  |  | Soil near Kebena River (Embassy of Italy area) ((KIS) | KIS |
| 11 | Bantyiketu River (BYR) | Bantyiketu River discrete (BRD) | BRD |
|  |  | Bantyiketu River mixed (BRM) | BRM |
|  |  | Soil near Bantyiketu River (BRS) | BRS |
| 12 | Gedelgibu + Kebena Joint (GGKR) | Gedelgibu + Kebena joint discrete (GKD) | GKD |
|  |  | Gedelgibu + Kebena joint mixed (GKM) | GKM |
|  |  | Soil near Gedelgibu + Kebena joint (GKS) | GKS |
| 13 | Harbu River (HR) | Abado River discrete (HRD) | HRD |
|  |  | Abado River mixed (HRM) | HRM |
|  |  | Soil near Abado River (HRS) | HRS |
| 14 | Little Akaki River (LAR) | Little Akaki River discrete (LAD) | LAD |
|  |  | Little Akaki River mixed (LAM) | LAM |
|  |  | Soil Little Akaki River (LAS) | LAS |
| 15 | Jemo River (JR) | Jemo river Discrete (JMD) | JMD |
|  |  | Jemo River Mixed (JMM) | JMM |
|  |  | Soil near Jemo River (JMS) | JMS |
| 16 | Saris Area Wastewater (SAW) | Saris area wastewater discrete (SWD) | SWD |
|  |  | Saris area wastewater mixed (SWM) | SWM |
|  |  | Soil near Saris area wastewater (SWS) | SWS |
| 17 | Gerji area wastewater (GAW) | Gerji area wastewater discrete (GWD) | GWD |
|  |  | Gerji area waste water mixed (GWM) | GWM |
|  |  | Soil near Gerji area wastewater (GWS) | GWS |
| 18 | Yekatit 12 Hospital (Y12H) | Yekatit 12 Hospital sewage discrete (YHD) | YHD |
|  |  | Yekatit 12 Hospital sewage mixed (YHM) | YHM |
|  |  | Soil from Yekatit 12 Hospital (YHS) | YHS |
| 19 | Menelik II Hospital (MIIH) | Menelik II Hospital sewage discrete (MHD) | MHD |
|  |  | Menelik II Hospital sewage mixed (MHM) | MHM |
|  |  | Soil from Menelik II Hospital (MHS) | MHS |
| 20 | Zewditu Memorial Hospital | Zewditu Memorial Hospital sewage discrete (ZHD) | ZHD |
|  |  | Zewditu Memorial Hospital sewage mixed (ZHM) | ZHM |
|  |  | Soil from Zewditu Memorial Hospital (ZHS) | ZHS |
