# Supplementary material for: Isolation, purification, and phenotypic characterization of virulent Klebsiella pneumoniae phages from environmental samples in Addis Ababa, Ethiopia: A synergistic approach combining spot assay and streak plating
Source: PLoS One. 2025 Sep 24;20(9):e0331955. doi: 10.1371/journal.pone.0331955 (PMC12459788; doi:10.1371/journal.pone.0331955)
Supplement: S2 Table — (DOCX) [file pone.0331955.s004.docx]

**Table 2. List of bacteria isolates used in this study**

| **S.no** | **Isolate code** | **Bacteria** | **Source** | **Use** |
| --- | --- | --- | --- | --- |
| 1 | ®ATCC700603 | *K. pneumoniae* | EPHI | HR |
| 2 | ®ATCC25922 | *E. coli* | EPHI | HR |
| 3 | ®27853 | *P. aeruginosa* | EPHI | HR |
| 4 | Ab14 | *A. baumannii* | EPHI | HR |
| 5 | Pm06 | *P. mirabilis* | EPHI | HR |
| 6 | Kp05 | *K. pneumoniae* | EPHI | HR |
| 7 | Kp02 | *K. pneumoniae* | EPHI | HR |
| 8 | Kp03 | *K. pneumoniae* | EPHI | HR |
| 9 | Kp04 | *K. pneumoniae* | EPHI | HR |
| 10 | Kp01 | *K. pneumoniae* | EPHI | HR |
| 11 | Kox01 | *K. oxytoca* | EPHI | HR |
| 12 | Kox02 | *K. oxytoca* | EPHI | HR |
| 13 | Koz01 | *K. ozaenae* | EPHI | HR |
| 14 | Koz02 | *K. ozaenae* | EPHI | HR |
| 15 | TA-SP04 | *K. pneumoniae* | IB-HBL | HR |
| 16 | TA-SP06 | *K. pneumoniae* | IB-HBL | **H** |
| 17 | TA-SP09 | *K. pneumoniae* | IB-HBL | **H** |
| 18 | TA-SP17 | *K pneumoniae* | IB-HBL | HR |
| 19 | TA-SP18 | *K. pneumoniae* | IB-HBL | **H** |
| 20 | TA-SP22 | *K. pneumoniae* | IB-HBL | **H** |
| 21 | TA-SP 23 | *K. pneumoniae* | IB-HBL | HR |
| 22 | TA-SP28 | *K. pneumoniae* | IB-HBL | HR |
| 23 | TA-SP32 | *K. pneumoniae* | IB-HBL | **H** |
| 24 | TA-SP37 | *K. pneumoniae* | IB-HBL | **H** |
| 25 | TA-SP41 | *K pneumoniae* | IB-HBL | HR |
| 26 | TA-SP45 | *K. pneumoniae* | IB-HBL | **H** |
| 27 | TA-SP50 | *K. pneumoniae* | IB-HBL | HR |
| 28 | TA-SP54 | *K. pneumoniae* | IB-HBL | HR |
| 29 | TA-SP59 | *K. pneumoniae* | IB-HBL | HR |
| 30 | TA-SP65 | *K. pneumoniae* | IB-HBL | HR |
| 31 | TA-SP72 | *K. pneumoniae* | IB-HBL | HR |
| 32 | TA-SP76 | *K. pneumoniae* | IB-HBL | **H** |
| 33 | TA-SP82 | *K. pneumoniae* | IB-HBL | HR |
| 34 | TA-SP-87 | *K. pneumoniae* | IB-HBL | HR |
| 35 | TA-SP88 | *K. pneumoniae* | IB-HBL | HR |
| 36 | TA-SP 92 | *K. pneumoniae* | IB-HBL | **H** |
| 37 | TA-SP96 | *K. pneumoniae* | IB-HBL | HR |
| 38 | TA-SP101 | *K. pneumoniae* | IB-HBL | H |
| 39 | TA-SP109 | *K. pneumoniae* | IB-HBL | HR |
| 40 | TA-SP110 | *K. pneumoniae* | IB-HBL | HR |
| 41 | TA-SP117 | *K. pneumoniae* | IB-HBL | **H** |
| 42 | TA-SP118 | *K. pneumoniae* | IB-HBL | HR |
| 43 | TA-SP121 | *K. pneumoniae* | IB-HBL | HR |
| 44 | TA-SP126 | *K. pneumoniae* | IB-HBL | HR |
| 45 | TA-SP129 | *K. pneumoniae* | IB-HBL | HR |

“**H**” Isolation Host”, “**HR**” Host Range, “**EPHI**” Ethiopian Public Health Institute, “**IB-HBL**” Institute of Biotechnology Health Biotechnology Laboratory.
